# Differential Associations of White Matter Brain Age With Language-Related Mechanisms in Word-Finding Ability Across the Adult Lifespan

**DOI:** 10.3389/fnagi.2021.701565

**Published:** 2021-09-03

**Authors:** Pin-Yu Chen, Chang-Le Chen, Hui-Ming Tseng, Yung-Chin Hsu, Chi-Wen Christina Huang, Wing P. Chan, Wen-Yih I. Tseng

**Affiliations:** ^1^Molecular Imaging Centre, National Taiwan University, Taipei, Taiwan; ^2^Institute of Medical Device and Imaging, National Taiwan University College of Medicine, Taipei, Taiwan; ^3^AcroViz Technology Inc., Taipei, Taiwan; ^4^Department of Radiology, Wan Fang Hospital, Taipei Medical University, Taipei, Taiwan; ^5^Department of Radiology, College of Medicine, Taipei Medical University, Taipei, Taiwan

**Keywords:** brain age prediction, white matter, diffusion MRI, word-finding ability, domain-specific language mechanisms

## Abstract

Research on cognitive aging has established that word-finding ability declines progressively in late adulthood, whereas semantic mechanism in the language system is relatively stable. The aim of the present study was to investigate the associations of word-finding ability and language-related components with brain aging status, which was quantified by using the brain age paradigm. A total of 616 healthy participants aged 18–88 years from the Cambridge Centre for Ageing and Neuroscience databank were recruited. The picture-naming task was used to test the participants’ language-related word retrieval ability through word-finding and word-generation processes. The naming response time (RT) and accuracy were measured under a baseline condition and two priming conditions, namely phonological and semantic priming. To estimate brain age, we established a brain age prediction model based on white matter (WM) features and estimated the modality-specific predicted age difference (PAD). Mass partial correlation analyses were performed to test the associations of WM-PAD with the cognitive performance measures under the baseline and two priming conditions. We observed that the domain-specific language WM-PAD and domain-general WM-PAD were significantly correlated with general word-finding ability. The phonological mechanism, not the semantic mechanism, in word-finding ability was significantly correlated with the domain-specific WM-PAD. In contrast, all behavioral measures of the conditions in the picture priming task were significantly associated with chronological age. The results suggest that chronological aging and WM aging have differential effects on language-related word retrieval functions, and support that cognitive alterations in word-finding functions involve not only the domain-specific processing within the frontotemporal language network but also the domain-general processing of executive functions in the fronto-parieto-occipital (or multi-demand) network. The findings further indicate that the phonological aspect of word retrieval ability declines as cerebral WM ages, whereas the semantic aspect is relatively resilient or unrelated to WM aging.

## Highlights

- Differential associations of white matter (WM) brain age with word-finding ability were observed.
- Domain-specific language system in the phonological component, but not the semantic component, was correlated with WM brain age.
- Word finding functions involve not only the domain-specific processing but also the domain-general processing of executive functions.
- WM brain age is a potential indicator of cognitive decline in language-related word-finding ability.

## Introduction

Older adults experience problems in daily word-finding tasks such as recalling precise names of people or objects (Labarge, 1986; Sunderland et al., 1986; Schmitter-Edgecombe et al., 2000). Such performance impairment manifests as slow efficiency, increased pauses during speech production, or being unable to remember phonology when intending to express a known word (Obler and Albert, 1981; Nicholas et al., 1985; Kemper et al., 1990; Cheung and Kemper, 1992; Orange and Purves, 1996). The increased word-finding difficulties constitute a major complaint among older adults.

Two potential cognitive mechanisms underlying these impairments in word retrieval and generation ability have been identified. The first posits that normal aging leads to a decreased ability in language-related ability such as the stored conceptual representations and lexical knowledge; the second posits that normal aging causes a general decline in the executive functions involved in retrieval mechanisms. Working memory and executive functions required for the word retrieval processes involve inhibitory control, lexical selection, and competition monitoring. Earlier studies on cognitive aging found that unlike other cognitive functions that are sensitive to aging, such as working memory and executive functions (Daniels et al., 2006; Dennis and Cabeza, 2008; Collette et al., 2009; Higby et al., 2019), language abilities (e.g., lexical knowledge and semantic representation) remain stable and even improve with age (Kave et al., 2009; Salthouse, 2009; Meyer and Federmeier, 2010; Verhaegen and Poncelet, 2013).

However, some researchers explained the causes of word retrieval failure from other aspects of language processes such as phonological processes (Taylor and Burke, 2002; Ouyang et al., 2020), an age-related decrease of connectivity in the language system (Burke et al., 1991) and stronger lexical competition (Lagrone and Spieler, 2006). Two potential theories have been developed to provide possible interpretations for the age-related decline in language processes. One which is called the inhibition deficit theory (IDT) suggests that the elderly show decreased control of the information processing and become more distracted in both semantic and phonological related mechanisms (Hasher et al., 1991). The other, called the transmission deficit hypothesis (TDH), states that the phonological mechanism weakens with age and results in word retrieval failure, but the semantic mechanism is preserved (Mackay and Burke, 1990).

When linking behavioral performance with neural activities, neuroimaging studies have supported the frontotemporal system as the neural substrate of language function (Van Der Lely and Pinker, 2014; Chang et al., 2015; and Lopez-Barroso and De Diego-Balaguer, 2017; Campbell and Tyler, 2018). Other studies have extended the domain-specific language network (LN) to a domain-general system or multi-demand network including the frontoparietal connections (Duncan, 1995; Unsworth et al., 2014; Cole et al., 2015; Duncan et al., 2017) and observed an association between the age-related decline of the domain-general system or multi-demand network and that of executive functions such as inhibition, attention, and working memory (Duncan et al., 2017; Chen P.-Y. et al., 2020).

Multiple neuroimaging studies have demonstrated that cognitive decline is related to the deterioration of brain structure and function (Archer et al., 2018; Fletcher et al., 2018). Previous studies examined the biological relationships of language and memory abilities with white matter (WM) tracts across adulthoods using diffusion magnetic resonance imaging (MRI) techniques (Ziegler et al., 2010; Stamatakis et al., 2011; Sexton et al., 2014; Feng et al., 2016; Houston et al., 2019). For example, the fronto-temporal link of the inferior fronto-occipital fasciculus (IFOF) might be involved in the figure-recall ability (Voineskos et al., 2012). The IFOF might also play a role in language function according to the investigation of intraoperative electrostimulation to this pathway (Duffau et al., 2005). It has been reported that the posterior projections of the corpus callosum might contribute to the domains in memory and executive function (Voineskos et al., 2012). These findings of varying relationships of different WM tracts with language and memory functions support that the integrity of structural connectivity may contribute to the performance of language and memory functions.

To our knowledge, the performance of word retrieval ability might alter along the dimension of chronological age, but it is unclear whether such ability is related to the biological age of brain structures. The growing body of neuroimaging studies has developed brain age prediction models that involve machine learning techniques to evaluate and quantify an individual’s status of brain aging (Cole and Franke, 2017; Franke and Gaser, 2019). The assumption underlying this approach is that the biological aging process of the brain in a cognitively normal population is chronological, so brain age prediction models are created by estimating the regression pattern between neuroimaging features and chronological age in a normative population. The established models are then applied to the cohort of interest to predict individuals’ brain age. The inference of brain age prediction models can be used to quantify the status of brain aging by calculating the discrepancy between the chronological and biological age of the brain (Smith et al., 2019). The difference between predicted age and chronological age is defined as the predicted age difference (PAD) of the brain, which represents the degree of deviation from the chronological patterns of brain aging in a normative population. The PAD can be regarded as a non-chronological age variable and has been employed to predict the integrity of brain structures, the increased risks of dementia, and the degree of domain-specific cognitive decline (Gaser et al., 2013; Boyle et al., 2020; Cole, 2020). Also, the PAD of WM (WM-PAD) could be used to detect aberrant brain aging in neurodegenerative diseases and psychiatric disorders (Chen et al., 2019, 2020c), and reflect the biological associations with several cognitive functions (Chen et al., 2020a). Employing the brain age paradigm, we aimed to test the hypothesis of word retrieval ability was associated with the biological age of WM.

**Figure 1:**
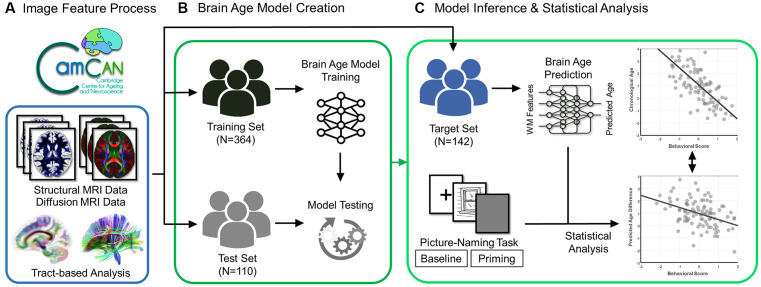
Flowchart of experiment design. The brain scans (structural and diffusion MRI data) and behavioral data (scores of the picture-naming task in baseline and priming conditions) were acquired from the participants in the Cam-CAN Repository. **(A)** The diffusion MRI data went through the tract-based analysis. **(B)** The processed data were used to create and test the brain age prediction model in the training and test sets, respectively. **(C)** Eventually, the established brain age model was applied to the target set to predict the value of the predicted age difference (PAD). The statistical analysis was further conducted to estimate the association of behavioral scores with chronological age and predicted age difference in the target set.

In the current study, we investigated whether the retrieval ability is a domain-specific language process or requires domain-general mechanisms incorporation, further we examined the association of semantic and phonological components in the language system involved in word-finding ability with the brain aging of WM. We employed the picture-naming task to evaluate word retrieval ability and the semantic and phonological mechanisms involved in a population-based lifespan adult cohort, and the brain age paradigm was used to assess the degree of WM aging. Figure 1 illustrates the experimental design of the present study. Compared to the relationship between cognitive decline and chronological age reported in previous studies (Stevens et al., 2008; Ward et al., 2013; Baciu et al., 2016), we hypothesized that the WM-PAD can differentiate whether the retrieval ability is a domain-specific language process or requires domain-general mechanisms incorporation and further manifest different associations patterns with semantic and phonological mechanisms of language system involved in word-finding ability. Our findings could advance the understanding of the biological aging effect of WM in the word-finding ability.

## Materials and Methods

### Participants

The participants were recruited from the Cambridge Center for Ageing and Neuroscience (Cam-CAN) project^1^. A detailed description of the recruitment process has been reported in the studies by Shafto et al. (2014) and Taylor et al. (2017). The East of England–Cambridge Central Research Ethics Committee approved the study protocol. After providing written informed consent, all participants underwent a diverse set of neuropsychological tests, cognitive tasks, and MRI scanning. Participants with a Mini-Mental State Examination score of 24 or lower, those with a poor fluency in English, those with poor vision or hearing, those with self-reported substance abuse, and those who were currently experiencing serious health problems were excluded. Participants who did not meet the safety and health-related criteria for MRI were also excluded.

A total of 616 participants aged 18–88 years were recruited. The participants were assigned to one of three groups, namely the training, test, and target groups. These three groups were used to create brain age prediction models, test model performance, and estimate brain age measures for statistical analyses of the experiments, respectively. Specifically, participants who had complete cognitive measurements were preferentially assigned to the target group. The remaining participants were split into the training and test groups through a conditional random approach to guarantee statistically identical age and sex distributions between the groups. Table 1 lists the demographic information of the participants in the three groups.

**Table 1:** Demographics and cognitive performance of the picture-naming task from healthy adults in the Cam-CAN cohort.

	Training group	Test group	Target group	One-way ANOVA
Subject number	364	110	142
Age mean (±SD)	54.18 (18.28)	54.55 (18.61)	54.23 (18.84)	*F* = 0.017, *p* = 0.983
Gender (M/F)	51.10/48.90	49.09/50.91	46.48/53.22
Education years (M/F)	14.60/14.22	15.24/13.98	14.42/15.04	*F* = 0.389, *p* = 0.678
Baseline accuracy (%)				
range	50–94	51–92	53–93	
Mean (±SD)	78 (9)	78 (8)	79 (8)	*F* = 0.578, *p* = 0.561
Baseline RT (millisecond)				
range	546–1138	643–1175	610–1056	
Mean (±SD)	817.87 (81.24)	819.65 (86.45)	809.35 (92.01)	*F* = 1.211, *p* = 0.299
Phonological primary accuracy (%)				
range	53–100	60–100	63–100	
Mean (±SD)	90 (9)	90 (9)	91 (8)	*F* = 0.340, *p* = 0.712
Phonological priming RT (millisecond)				
range	510–1164	541.5–1143	584–1160.5	
Mean (±SD)	803.25 (87.73)	788.41 (91.94)	789.12 (96.17)	*F* = 0.291, *p* = 0.748
Semantic priming accuracy (%)				
range	48–100	63–100	55–100	
Mean (±SD)	87 (9)	85 (18)	89 (8)	*F* = 1.113, *p* = 0.329
Semantic priming RT (millisecond)				
range	523–1,288	625–1,063	576.5–1,139	
Mean (±SD)	785.08 (90.11)	763.18 (169.87)	790.04 (92.43)	*F* = 0.012, *p* = 0.988

*Note: RT, response time*.

### Behavioral Task

The picture-naming task was used to test language-related explicit and implicit effects involving word finding and word generation. The task measured the naming response time (RT) and accuracy (ACC) under the baseline condition and two priming conditions (i.e., phonological and semantic priming). The participants were asked to name every picture as quickly and accurately as possible. The baseline condition required an individual to consciously recall language-related cognitive ability (explicit effect). It included 200 pictures of common objects presented in a pseudorandom order. The baseline experiment procedure consisted of a fixation point displayed for 500 ms, followed by the target picture displayed for 750 ms, and finally, a blank screen displayed for 1,000 ms. The priming condition probed an individual’s unconscious language-related ability (implicit effect), activated by specific cues. The priming condition consisted of one baseline picture preceded by a prime picture, which was unrelated, phonologically related, or semantically related to the baseline pictures. Each trial consisted of 500 ms fixation, followed by 750 ms of the prime picture and 1,000 ms blank screen, and proceeded by 750 ms of the target picture and 2,500 ms of a blank screen.

Phonologically related priming pairs included overlapping phonology (first two phonemes in English; e.g., glass–glove or first phonemes in English; e.g., ring–ruler). Semantically related priming pairs included semantic relationships (e.g., tiger–cat or cabinet–box). Unrelated pairs were neither semantically nor phonologically related. Table 1 provides the demographic information and cognitive performance on the picture-naming task of the healthy participants in the study. The paired *t*-tests of ACC and RT between primed and unrelated conditions were conducted with the estimation of the Cohen’s d for the effect size to evaluate the priming effects (Table 2). To evaluate the cognitive performance in the target group (*N* = 142), the correlation matrix approach adjusting chronological age and education was employed to examine the associations between the cognitive scores (i.e., ACC and RT) under each condition of tasks (Table 3). Cognitive performance on the picture-naming task was assessed for its correlation with the chronological age variable (Table 4).

**Table 2:** Results of paired *t*-test of ACC and RT between primed and unrelated conditions with Cohen’s d for effect size.

**ACC**	**Primed condition**	**Unrelated condition**	***T*-value**	***P*-value**	**Cohen’s d**
Phonological	0.907 (0.078)	0.876 (0.094)	*t*(141) = 4.66	*p* = 0.000	0.355
Semantic	0.886 (0.083)	0.876 (0.094)	*t*(141) = 1.38	*p* = 0.170	0.109
**RT**					
Phonological	789.1 (96.2)	808.8 (97.8)	*t*(141) = −2.90	*p* = 0.004	−0.203
Semantic	790.0 (92.4)	808.8 (97.8)	*t*(141) = −3.26	*p* = 0.001	−0.197

*Note: ACC, accuracy; RT, response time*.

**Table 3:** The correlation matrix between ACC/RT under each conditions of the tasks.

**ACC**	**Baseline**	**Phonological**	**Semantic**	**Unrelated**
*Baseline*	1.000	0.737	0.678	0.626
*Phonological*	0.737	1.000	0.568	0.512
*Semantic*	0.678	0.568	1.000	0.453
*Unrelated*	0.626	0.512	0.453	1.000
**RT**				
*Baseline*	1.000	0.579	0.605	0.636
*Phonological*	0.579	1.000	0.642	0.553
*Semantic*	0.605	0.642	1.000	0.674
*Unrelated*	0.636	0.553	0.674	1.000
∣rule
**Priming effect**	**Phonological—unrelated**		**Semantic—Unrelated**	
**ACC**		
*Phonological—Unrelated*	1.000		0.670	
*Semantic—Unrelated*	0.670		1.000	
**RT**		
*Phonological—Unrelated*	1.000		0.605	
*Semantic—Unrelated*	0.605		1.000	

*Note: age and education as covariates. All *p*-values <0.001. ACC, accuracy; RT, response time*.

**Table 4:** Partial correlations (*r*) between cognitive performances (ACC and RT) of the picture naming task with chronological age and brain age.

**Measures**	**ACC**		**RT**	
**Chronological Age**		
*Baseline*	*r* = −0.546	*p* < 0.001**	*r* = 0.438	*p* < 0.001**
*Phonological*	*r* = −0.397	*p* < 0.001**	*r* = 0.554	*p* < 0.001**
*Semantic*	*r* = −0.452	*p* < 0.001**	*r* = 0.522	*p* < 0.001**
*Unrelated*	*r* = −0.408	*p* < 0.001**	*r* = 0.426	*p* < 0.001**
**PAD—Whole Brain**		
*Baseline*	*r* = −0.236	*p* < 0.005**	*r* = 0.019	*p* < 0.827
*Phonological*	*r* = −0.200	*p* < 0.018*	*r* = 0.009	*p* < 0.915
*Semantic*	*r* = −0.071	*p* < 0.401	*r* = 0.056	*p* < 0.514
*Unrelated*	*r* = −0.101	*p* < 0.234	*r* = 0.076	*p* < 0.375
**PAD—Language Network**		
*Baseline*	*r* = −0.285	*p* < 0.001**	*r* = 0.020	*p* < 0.818
*Phonological*	*r* = −0.178	*p* < 0.035*	*r* = 0.099	*p* < 0.242
*Semantic*	*r* = −0.116	*p* < 0.171	*r* = 0.103	*p* < 0.227
*Unrelated*	*r* = −0.127	*p* < 0.135	*r* = 0.018	*p* < 0.830
**PAD—Non-Language Regions**		
*Baseline*	*r* = −0.203	*p* < 0.016*	*r* = 0.014	*p* < 0.874
*Phonological*	*r* = −0.146	*p* < 0.085	*r* = 0.008	*p* < 0.921
*Semantic*	*r* = −0.055	*p* < 0.516	*r* = 0.024	*p* < 0.775
*Unrelated*	*r* = −0.048	*p* < 0.573	*r* = −0.016	*p* < 0.847

Note: The partial correlation approach was adopted to examine the associations of PAD measures with the cognitive indices (ACC and RT) while chronological age and education factors were controlled. Only education factor was controlled when estimating correlations of chronological age with cognitive indices. ACC, accuracy; RT, response time; PAD, predicted age difference; * = marginal correlation, *p* < 0.05; ** = significant correlation, *p* < 0.0125 (adjusting for multiple comparison).

### Image Acquisition

All participants were scanned using a 3-Tesla MRI scanner (TIM–Trio, Siemens, Erlangen, Germany) at the Medical Research Council Cognition Brain and Sciences Unit, Cambridge, United Kingdom. High-resolution structural MR images were acquired using a three-dimensional rapid acquisition gradient-echo sequence with preparation pulses for T1-weighted contrast. The following imaging parameters were used: TR/TE = 2,250/2.99 ms, inversion time = 900 ms, flip angle = 9°, field of view_(FOV)_ = 256 × 240 × 192 mm^3^, resolution = 1 mm isotropic, and acquisition time of approximately 4.5 min. Diffusion-weighted images were acquired using a spin-echo sequence with two refocusing pulses to minimize distortion induced by eddy currents. The acquisition scheme entailed 30 diffusion gradient directions for each of the two diffusion sensitivity values (*b*-value) of 1,000 and 2,000 s/mm^2^ and three images with a *b*-value of 0 s/mm^2^. The imaging parameters were as follows: TR/TE = 9100/104 ms, FOV = 192 × 192 mm^2^, voxel size = 2 mm isotropic, 66 axial slices, number of averages = 1, and acquisition time = approximately 10 min.

### Quality Assurance of Diffusion-Weighted Images

All diffusion data sets underwent quality assurance procedures, including examinations of the signal-to-noise ratio (SNR), the degree of alignment between T1-weighted images and the GFA map, and the detection of abrupt head motion. SNR was evaluated through the calculation of the mean signal of an object divided by the SD of the background noise. In practice, the signal was determined using a central square of an image for each slice, and the noise from four corner regions was averaged. Diffusion data sets were included in the study if they had an SNR higher than the mean SNR minus 2.5 SDs of all participants. The degree of within-participant alignment between T1-weighted images and the GFA map was evaluated through the calculation of the spatial correlation between the WM tissue probability map derived from T1-weighted images and the GFA map. Data sets with correlation coefficients higher than the mean spatial correlation minus 2.5 SDs were included in the study. Instances of abrupt head motion or other visible artifacts were manually identified and removed. The correction algorithm for motion and eddy currents was used to detect and replace slices affected by signal loss due to bulk motion during acquisition (Andersson and Sotiropoulos, 2016). Of the initial 636 images, 616 (96.9%) underwent the quality assurance procedures and qualified for subsequent analyses.

### Diffusion Index Estimation Using Regularized Mean Apparent Propagator Algorithm

We used regularized mean apparent propagator (ReMAP) MRI algorithm to reconstruct the diffusion propagator from the diffusion MRI signal (Ozarslan et al., 2013; Hsu and Tseng, 2018). ReMAP MRI fits the diffusion MRI signal with a linear combination of Hermite functions so that the diffusion propagator can be represented by a few coefficients, serving as an efficient dimension reduction method. The coefficients of the diffusion propagator, orientation distribution function (ODF), and diffusion tensor were determined, from which various diffusion indices could be calculated. In this study, we calculated generalized fractional anisotropy (GFA) and mean diffusivity (MD) to represent the microstructural integrity of WM tracts. The GFA value was the standard deviation (SD) of the ODF in all radial directions divided by the norm of the ODF (Tuch, 2004). The value of MD was the mean of the first, second, and third eigenvalues of the diffusion tensor. GFA changes in response to the strength of anisotropic diffusion in the microstructure. MD is the diffusion index that is the most sensitive to age, and it has been considered useful for the prediction of brain age (Cox et al., 2016; Chen et al., 2020b).

### Tract-Based Analysis for Diffusion-Weighted Images

**Figure 2:**
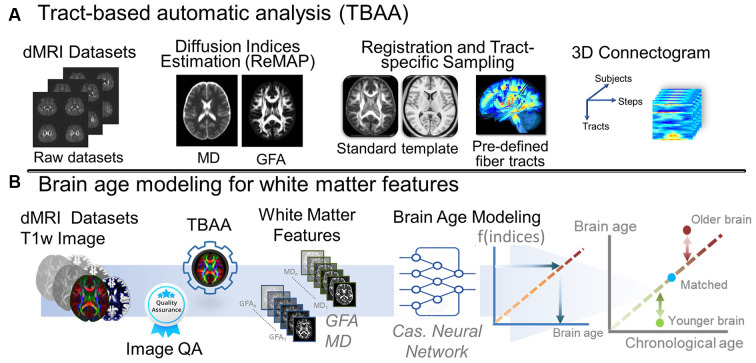
Analytic pipeline of diffusion MRI reconstruction and the corresponding brain age modeling for white matter features. **(A)** The pipeline depicts the pipeline of TBAA. **(B)** The pipeline displays the brain age modeling for WM. Abbreviation: dMRI, diffusion magnetic resonance imaging; MD, mean diffusivity; GFA, generalized fractional anisotropy; QA, quality assurance; Cas, cascade; ReMAP, regularized mean apparent propagator reconstruction.

We used an in-house analytic method, tract-based automatic analysis (TBAA; Chen et al., 2015), to sample the diffusion index values along the tracts across major WM regions. Figure 2A illustrates the entire process of diffusion index computation and tract-based analysis. In brief, TBAA was used to register diffusion-weighted images of all study participants to a standard template (Hsu et al., 2015), in which tractograms of 76 major WM tracts were developed using deterministic tractography. After registration was complete, the position coordinates of 76 tracts were transformed from the template space to the native space of each participant. Diffusion index values of GFA and MD were sampled along with the coordinates of each tract and averaged to obtain the mean GFA and MD indices for each WM tract. Subsequently, 76 GFA and 76 MD values were obtained from each participant as features for brain age estimation.

### Brain Age Prediction Based on WM Features Using Machine Learning

To estimate the brain age index of the participants, we established brain age prediction models based on the WM features from the training set. To differentiate biological underpinnings of language-related cognitive ability, we created three types of brain age models: models derived from features of the whole brain (WB), language network (LN), and non-language regions (NLR). The WB brain age model provided brain age metrics based on entire WM features (i.e., 76 GFA and 76 MD values). The LN brain age model was built based on the features defined in the language network (Van Der Lely and Pinker, 2014; Chang et al., 2015; Lopez-Barroso and De Diego-Balaguer, 2017); there were 14 tract bundles on language network by our definition, so the LN brain age metrics were estimated using 14 GFA and 14 MD values. Table 5 provides the constitution of the language network in WM. The NLR brain age model was created using the features which were not defined in the language network (i.e., 62 GFA and 62 MD values). Also, all three brain age models contained sex variables as another predictor. In practice, the brain age models were designed to have a 12-layer feedforward cascade neural network, and the related information of model design can be found in our previous study (Chen et al., 2020b). A 10-fold cross-validation procedure was performed to estimate the performance of the brain age model in the training phase. Next, an independent test set was used to evaluate the generalizability of the models. The modeling procedures were implemented using MATLAB R2019a (MathWorks Inc., Natick, MA, USA) with an NVIDIA GeForce RTX 2080Ti (NVIDIA Inc., Santa Clara, CA, USA) graphics processing unit for accelerated computing. The technical details of brain age prediction are provided in the Supplementary Material. The model performance was evaluated according to the Pearson correlation coefficient (*Rho*) and mean absolute error (MAE) between the predicted age and chronological age. Figure 2B displays the brain age modeling for WM.

**Table 5:** Fiber tract bundles of interest in the language network.

**Name of fiber tract bundles**	**Connected region 1**	**Connected region 2**
L AF	L inferior frontal gyrus opercular part	L superior temporal gyrus
R AF	R inferior frontal gyrus opercular part	R superior temporal gyrus
L IFOF	L orbitofrontal gyrus	Occipital lobe
R IFOF	R orbitofrontal gyrus	Occipital lobe
L SLF III	L inferior frontal gyrus opercular part	L angular gyrus
R SLF III	R inferior frontal gyrus opercular part	R angular gyrus
L UF	L orbitofrontal gyrus	L superior temporal pole
R UF	R orbitofrontal gyrus	R superior temporal pole
CC of VLPFC	L inferior frontal gyrus + part of middle frontal gyrus	R inferior frontal gyrus + part of middle frontal gyrus
CC of SMA	L supplementary motor areas	R supplementary motor areas
CC of superior temporal gyrus	L superior temporal gyrus	R superior temporal gyrus
CC of splenium	L occipital lobe	R occipital lobe
CC of genu	L orbitofrontal gyrus	R orbitofrontal gyrus
CC of inferior parietal lobule	L inferior parietal lobules	R inferior parietal lobules

*Abbreviations: AF, arcuate fasciculus; CC, corpus callosum; IFOF, inferior frontal-occipital fasciculus; L, left; R, right; SLF, superior longitudinal fasciculus; SMA, supplementary motor area; UF, uncinate fasciculus; VLPFC, ventrolateral prefrontal cortex*.

### Partial Correlation Analysis of Brain Age Measures With Behavioral Task Performance

Partial correlation analysis was employed to address the relationship between age measures (i.e., chronological age and brain age) and language-related cognitive abilities in the target group (*N* = 142). To quantify the extent of an individual’s brain aging, the PAD was calculated by subtracting chronological age from predicted age (i.e., predicted age − chronological age) to represent the biological age of the brain. In this experiment, we estimated three PAD measures, namely PAD-WM, PAD-LN, and PAD-NLR. Notably, the PAD was correlated with chronological age in the training phase, which represents a statistical bias in regression estimation (Smith et al., 2019). To minimize this bias, the partial correlation approach adjusting chronological age and education was employed to examine the associations of the PADs with the cognitive scores (i.e., ACC and RT) under each condition of tasks. In contrast, the partial correlation analysis only adjusted the education factor when estimating the correlation of cognitive scores with chronological age (Table 4). The Bonferroni correction was used to address multiple comparison problems.

## Results

### Brain Age Prediction Model

The prediction models estimated participants’ brain age with acceptable performance in the training, test, and target groups. For the WB model, the model performance was *Rho* = 0.951 and MAE = 4.53 years in the training group (*N* = 364), *Rho* = 0.907 and MAE = 6.61 years in the test group (*N* = 110), and *Rho* = 0.910 and MAE = 6.11 years in the target group (*N* = 142). For the LN model, the model performance was *Rho* = 0.905 and MAE = 6.13 years in the training group, *Rho* = 0.802 and MAE = 8.68 years in the test group, and *Rho* = 0.831 and MAE = 7.74 years in the target group. For the NLR model, the model performance was *Rho* = 0.955 and MAE = 4.33 years in the training group, *Rho* = 0.904 and MAE = 6.72 years in the test group, and *Rho* = 0.903 and MAE = 6.57 years in the target group. Besides the WB model, we observed that the NLR model provided better prediction than the LN model. This might result from the number of features employed by the NLR brain age model. The age-related bias in the brain age estimation was minimized by adjusting chronological age in the statistical analyses.

### Cognitive Performance and Its Priming Effect

Before conducting correlation analysis, additional paired *t*-tests were used to examine if the primed condition was faster than the unrelated condition in the target group (*N* = 142; Table 2). Cohen’s d was also reported as the strength of the priming effect. Under the primed condition, the RTs were 789.1 (96.2) ms and 790.0 (92.4) ms in the phonological and semantic conditions, respectively. These RTs were significantly shorter (*p* = 0.004 and 0.001, respectively) than those in the unrelated condition, which was 808.8 (97.8) ms. In addition, the same statistical tests were applied to the measures of accuracy. We found that the accuracy at the phonological condition [(mean: 0.907, SD: (0.078)] was significantly better (*p* < 0.001) than that at the unrelated condition [0.876 (0.094)]; however, the accuracy at the semantic condition [0.886 (0.083)] was comparable to (*p* = 0.170) that at the unrelated condition.

To evaluate the cognitive performance in the target group (*N* = 142), the correlation matrix approach adjusting chronological age and education was employed to examine the associations between the cognitive scores (i.e., ACC and RT) under each condition of tasks (Table 3). All *p*-values were lower than 0.001. Both ACC and RT showed significant positive correlations across the task conditions but baseline ACC showed stronger linear relationships with all the tasks than baseline RT. Unrelated RT showed stronger linear relationships with all the tasks than unrelated ACC. For the correlation matrix examining the associations between the priming effects, each pair across ACC and RT represented similar linear relationships.

### Correlation Between Behavioral Task Performance on the Picture-Naming and Chronological Age

The performance (measured according to ACC and RT) of participants in the target group (*N* = 142) under the baseline and priming conditions in the picture-naming task was significantly correlated with their chronological age (Table 4). ACC showed significant negative correlations (*p* < 0.001) with chronological age under the baseline and priming conditions, whereas RT exhibited significant positive correlations (*p* < 0.001). Therefore, the chronological age variable was a strong factor influencing participants’ performance on the picture-naming task.

### Partial Correlations Between the WM PAD and Behavioral Task Performance on the Picture-Naming Task

**Figure 3:**
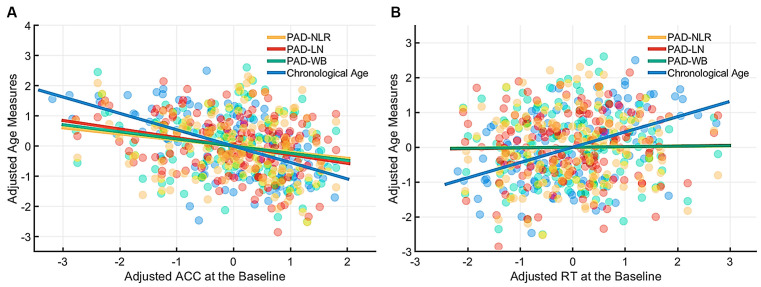
Partial correlation of cognitive indices (ACC and RT) at the baseline condition with age measures (chronological age and brain age). The brain age measures were significantly correlated with ACC under the baseline condition **(A)** but not with RT. **(B)** The lines of PAD (NLR, LN, and WB) were almost overlapped. Abbreviation: ACC, accuracy; RT, response time; PAD, predicted age difference; NLR, non-language regions; LN, language network; WB, whole brain.

**Figure 4:**
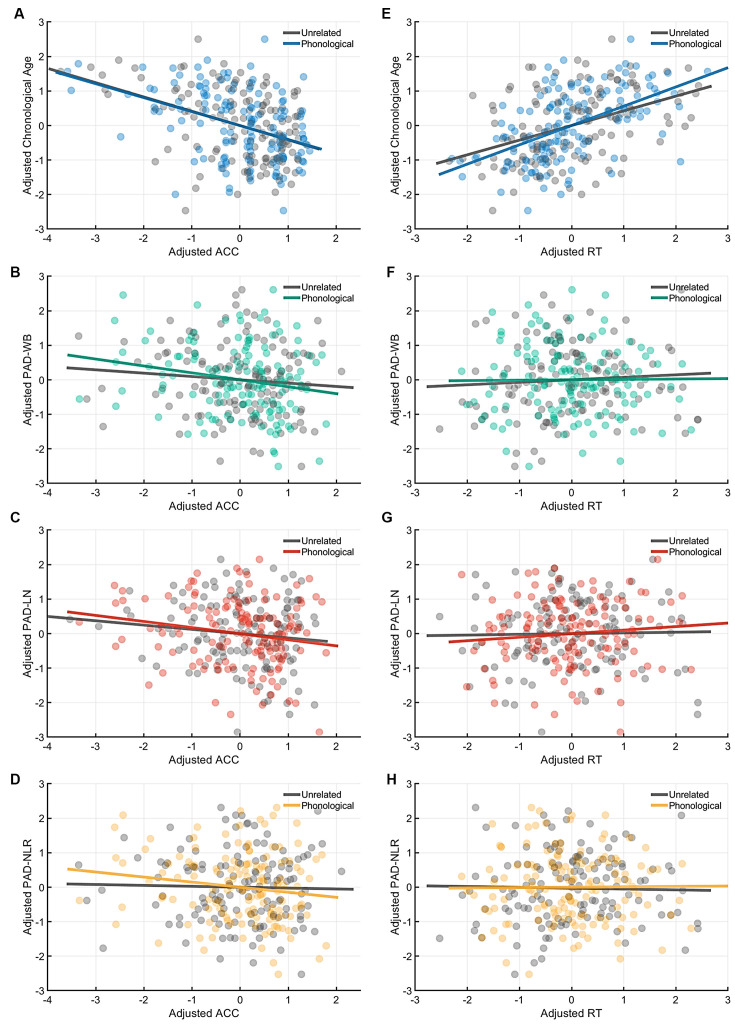
Partial correlation of the cognitive indices (ACC and RT) at the phonological-priming condition with age measures (chronological age and brain age). Under the phonological-related priming condition, chronological age was significantly correlated with ACC **(A)**, and the PAD-WB and PAD-LN were marginally associated with ACC. **(B–C)** However, the PAD-NLR was not associated with ACC. **(D)** On the other hand, only chronological age was significantly correlated with RT **(E)**, whereas all brain age measures were not correlated with RT **(F–H)**. Abbreviation: ACC, accuracy; RT, response time; PAD, predicted age difference; NLR, non-language regions; LN, language network; WB, whole brain.

**Figure 5:**
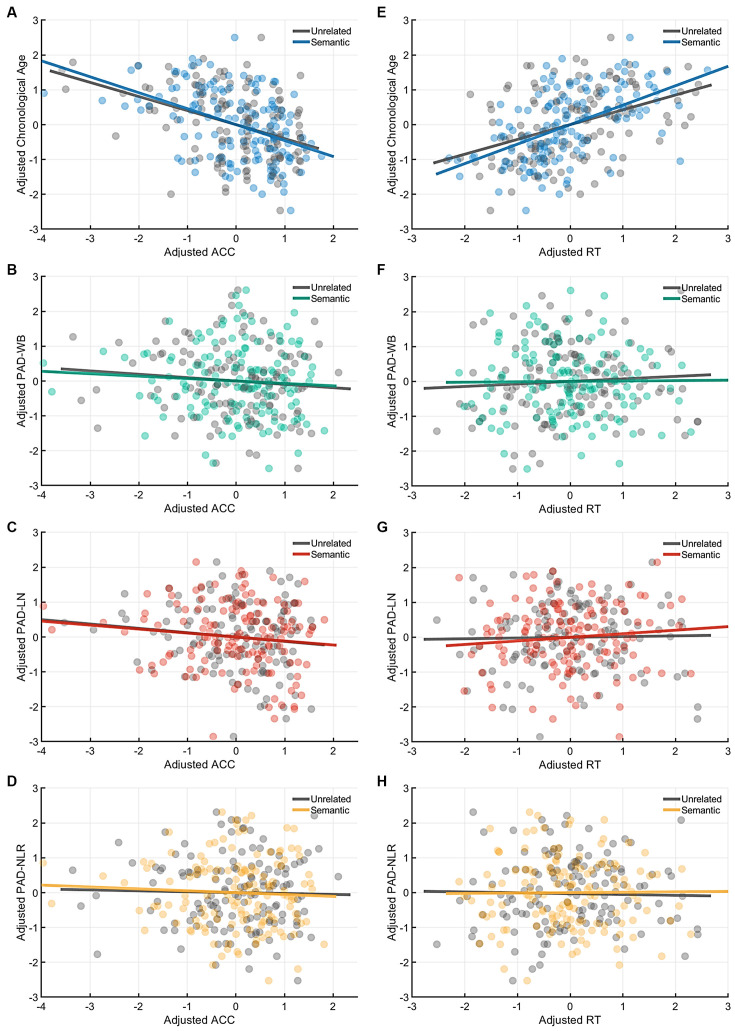
Partial correlation of the cognitive indices (ACC and RT) at the semantic-priming condition with age measures (chronological age and brain age). Under the semantic-related priming condition, chronological age was significantly correlated with ACC **(A)**, but no brain age measures were associated with ACC. **(B–D)** Likewise, only chronological age was significantly correlated with RT **(E)**, whereas all brain age measures were not correlated with RT **(F–H)**. Abbreviation: ACC, accuracy; RT, response time; PAD, predicted age difference; NLR, non-language regions; LN, language network; WB, whole brain.

Table 4 presents the partial correlations (*r*) between the cognitive indices (ACC and RT) in the picture-naming tasks and PAD measures of the target group (*N* = 142), and Figures 3–5 provide the results. The PAD-WB was significantly correlated with ACC under the baseline condition (*r* = −0.236, *p* = 0.005) but not with RT (*r* = 0.019, *p* = 0.827). Under the phonological-related priming condition, the PAD-WB was marginally associated with ACC (*r* = −0.200, *p* = 0.018; the Bonferroni-corrected threshold *α* = 0.0125) but not with RT (*r* = 0.009, *p* = 0.915). Similarly, the PAD-LN was significantly correlated with ACC under the baseline condition (*r* = −0.285, *p* = 0.001) but not with RT (*r* = 0.020, *p* = 0.818). Under the phonological-related priming condition, the PAD-LN was also marginally associated with ACC (*r* = −0.178, *p* = 0.035; the Bonferroni-corrected threshold *α* = 0.0125) but not with RT (*r* = 0.099, *p* = 0.242). However, the PAD-NLR was only marginally associated with ACC at the baseline condition (*r* = −0.203, *p* = 0.016). Under either the semantic-related priming or unrelated condition, all PAD measures (i.e., WB, LN, and NLR) did not exhibit significant associations with ACC or RT (Table 4).

## Discussion

To clarify the role of biological age of WM in word-finding ability, we investigated the associations of word-finding ability including semantic and phonological priming in language system with WM brain age; we tested the partial correlation between the performance indices (ACC and RT) in the picture-priming task and the language network, non-language network and whole brain of WM-PAD while adjusting for chronological age and education. Our results revealed two major findings: First, all WM-PADs in the language-network, non-language network, and over the whole-brain, which represented whole cerebral WM features instead of being confined to the frontotemporal language network, exhibited statistical associations with ACC in word retrieval functions while controlling the effect of chronological age. Such findings imply that cognitive alterations in word-finding functions involve not only the domain-specific language processing within the frontotemporal language network but also the domain-general processing of executive functions in the fronto-parieto-occipital (or multi-demand) network (Diachek et al., 2020). Second, the phonological priming ACC in the picture-naming task was associated with the language-network WM-PAD, whereas the semantic priming ACC in the picture-naming task exhibited no significant correlations. Chronological age was significantly associated with all conditions in the picture-priming task. The results suggest that for word-finding ability, the semantic mechanism in the language system might be more preserved than the phonological-related mechanism in the language system for the biological aging of WM. The findings further indicate that the phonological aspect of language slightly declines with increases in WM brain age, whereas the semantic aspect is relatively resistant or unrelated to WM aging.

## Brain Age Vs. Chronological Age in Word-Finding Ability

Multiple studies on cognitive aging have evaluated the association between cognitive functions and chronological age (Mackay and Burke, 1990; Hasher et al., 1991; Burke and Shafto, 2004; Lagrone and Spieler, 2006). Research on cognitive aging has established that the ability to successfully retrieve proper words from stored knowledge and concepts in long–term memory progressively declines in late adulthood (Christensen et al., 1997; Davis et al., 2001; Fleischman et al., 2004). The impaired word-generation ability in older individuals may attribute to the decline in retrieval mechanism of executive functions instead of impaired semantic representation (Fisk and Sharp, 2004; Waters and Caplan, 2005; Daniels et al., 2006; Dennis and Cabeza, 2008; Collette et al., 2009; Baciu et al., 2016; Higby et al., 2019). In our results, the ACC in baseline condition in the picture-priming task was significantly negative-correlated with the WM-PAD across the language network, non-language network, and whole-brain (Table 4). The findings show that the general word-finding ability reflected by the baseline condition of the picture-priming task not only requires support from the domain-specific language network but the broad domain-general system as well. The finding supports the previous research that the word retrieval mechanism involves language-related processes such as searching for linguistic information that would help assess the target, it also interacts among attention and executive functions of the domain-general system such as monitoring and inhibition to suppress competing representations and enhance the efficient retrieval from the long–term memory (Nozari et al., 2016; Nozari and Novick, 2017; Higby et al., 2019).

## WM Brain Age and Language-Related Mechanisms in Word-Finding Ability

In this study, ACC for phonological priming conditions showed a negative correlation with both the language-network WM-PAD and whole-brain WM-PAD but no significant relationship with non-language network WM-PAD (Table 4), meaning that the decline of ACC for phonological priming was associated with a higher WM-PAD and the phonological priming focuses on domain-specific language system. Older adults experience difficulties in retrieving and producing proper words because of weakening processes related to the phonological aspects of language ability (Taylor and Burke, 2002; Ossher et al., 2013; Rizio et al., 2017). Our results support those of earlier studies and further demonstrate that the biological relationship of WM brain age with the phonological mechanism in the language system occurs under conditions of phonological priming. The results imply that phonological priming might be affected by the biological aging of WM.

Contrary to the phonological component in the language system, the semantic component exhibited no significant linear relationships with WM-PAD. Previous behavioral studies have revealed that semantic processes, conceptual knowledge, and information are preserved during normal aging (Waters and Caplan, 2005; Kave et al., 2009; Salthouse, 2009; Meyer and Federmeier, 2010; Verhaegen and Poncelet, 2013). Multiple studies on cognitive aging have reported that language abilities such as lexical knowledge and semantic representation remain stable and even improve with age (Kave et al., 2009; Salthouse, 2009; Meyer and Federmeier, 2010; Verhaegen and Poncelet, 2013). The discrepancy between phonological and semantic components might implicate that, in the normal aging process, the biological effect of WM affects the retrieval ability of executive functions and word production speed more than the loss of conceptual or lexical knowledge (semantic component). The current finding supports the transmission deficit hypothesis (TDH) stating the phonological mechanism weakens with age and results in word retrieval failure, but the semantic mechanism is preserved (Mackay and Burke, 1990). TDH assumed that the semantic function requires multiple connections between the lexical component of a word and its semantic representation. and the multiplicity of connections may make it resistant to word-finding failure. In contrast, the phonological representation shows one-to-one mapping to the lexical component of a word, so it becomes vulnerable to aging (Burke and Shafto, 2004).

Previous studies have reported a chronological age-related decline in word-finding ability reflected by reduced retrieval speed (RT) or ACC (Stevens et al., 2008; Rizio et al., 2017). Consistently, we found that both ACC and RT were significantly correlated with chronological age. However, by using the WM brain age instead, our results showed that the WM-PAD was significantly associated with the cognitive measures in ACC but not in RT. This implicates that ACC and RT might reflect distinct dimensions of word-finding capacity; the ACC index reflected an individual’s retrieval precision, and the RT index manifested an individual’s retrieval efficiency. This discrepancy in the current study implies that the WM-PAD may not fully explain the word retrieval efficiency measured by RT in the picture-priming task.

In contrast to the effect of chronological age, neuroimaging-based brain age measures can reflect the differential trajectory against chronological age that might attribute to individual variations in domain-specific cognitions (Cole et al., 2018) and represent the status along the dimension of biological aging. Our study investigated the domain-specific association between language-related mechanisms in word retrieval processes and WM aging (i.e., WM-PAD), and the results showed that the language components that participated in word-finding ability were correlated with the biological age of WM, suggesting that the effect of WM aging might selectively contribute to the changes in word-finding ability. These findings provided the genuine associations of WM aging with language-related mechanisms in word retrieval processes and could advance our understanding of differentiating the effects between chronological aging and biological aging in WM on general word-finding ability and further the language-related mechanisms in word retrieval processes.

## Limitations

This study has some limitations. First, the picture-naming task adopted to assess word-finding ability in the current study is one of many cognitive tasks. Various tasks are used to assess cognitive functions, and a single task cannot fully assess the performance of complex cognitive functions such as language and memory. The picture-naming task, including the baseline and priming conditions, focuses on testing the abilities of conceptual recognition and word generation. The design of the picture priming task in our study did not include an encoding phase and the design of the priming conditions did not meet the conventional design of repetition priming which includes the encoding and the testing phase. Nevertheless, the present findings represent only a limited representation of cognitive aging involving language-related priming and word retrieval mechanism. Exploration of the associations of brain age with other cognitive tasks would provide a more complete picture of the brain–cognition relationships in cognitive aging. Second, the brain age models that we developed might not be optimal. For WM brain age, we used GFA and MD only on 76 major fiber tracts as our features of machine learning. We did not explore the whole spectrum of diffusion indices such as axial diffusivity, radial diffusivity, kurtosis, or neurite density, each of which represents distinct characteristics of WM microstructure. Adding some of the indices to brain age modeling might improve its association with cognitive aging. In this study, we built the brain age model using WM features over the entire brain. The null results for WM brain age might be attributed to the fact that the 76 major fiber tracts do not include the small fibers or *U* fibers in the brain because of limitations of the diffusion MRI technology used in this study. Therefore, the WM features in the present study do not capture the full scope of WM changes. Further studies are required to develop various brain age models to clarify the relationship between brain aging and cognitive aging.

## Conclusion

By investigating the associations between the PAD and task indices of memory functions, we revealed the genuine effect of WM aging on word-finding ability and its language-related mechanisms regardless of the chronological age effect. All behavioral measures of picture priming tasks were associated with chronological age, whereas only general word retrieval ability and the phonological-related word-finding ability were correlated with WM brain age. The results suggest that chronological aging and brain aging have differential effects on word retrieval functions; additionally, a new paradigm is introduced to investigate brain correlates of cognitive aging using brain age modeling.

## Data Availability Statement

The datasets presented in this study can be found in online repositories. The names of the repository/repositories and accession number(s) can be found below: https://www.cam-can.org/index.php?content=dataset.

## Ethics Statement

The studies involving human participants were reviewed and approved by The East of England–Cambridge Central Research Ethics Committee. The patients/participants provided their written informed consent to participate in this study.

## Author Contributions

P-YC: conceptualization, writing—original draft. C-LC: writing—original draft, formal analysis, and visualization. H-MT: formal analysis and visualization. Y-CH: software. W-YT: conceptualization, writing—original draft, and resources. All authors contributed to the article and approved the submitted version.

## Conflict of Interest

Y-CH was employed by the company AcroViz Technology Inc., Taipei, Taiwan. The remaining authors declare that the research was conducted in the absence of any commercial or financial relationships that could be construed as a potential conflict of interest.

## Publisher’s Note

All claims expressed in this article are solely those of the authors and do not necessarily represent those of their affiliated organizations, or those of the publisher, the editors and the reviewers. Any product that may be evaluated in this article, or claim that may be made by its manufacturer, is not guaranteed or endorsed by the publisher.

## Footnotes

^1^ http://www.cam-can.com
