# Supplementary material for: Differential Associations of White Matter Brain Age With Language-Related Mechanisms in Word-Finding Ability Across the Adult Lifespan
Source: Front Aging Neurosci. 2021 Sep 3;13:701565. doi: 10.3389/fnagi.2021.701565 (PMC8446673; doi:10.3389/fnagi.2021.701565)
Supplement: Supplementary file 1 [file Data_Sheet_1.PDF]

Supplementary document

S1 Brain Age Prediction

S1.1 Brain Age Modeling

In this study, 616 participants aged 18 to 88 years were assigned to one of three groups, namely the training, test, and target groups. These three groups were used to create brain age prediction models, to test model performance, and to estimate brain age measures for statistical analyses of the experiments, respectively. Specifically, participants who had complete cognitive measurements were preferentially assigned to the target group. The remaining participants were split into the training and test groups through a conditional random approach to guarantee statistically identical age and sex distributions between the groups (training set [N = 364]: mean age = 54.18 [18.28] years, max = 88, min = 18, female proportion = 48.9%; test set [N = 110]: mean age = 54.55 [18.61] years, max = 88, min = 18, female proportion = 50.91%). All participants were scanned using a 3-Tesla MRI scanner (TIM–Trio, Siemens, Erlangen, Germany) to obtain high-resolution T1-weighted images and two-shell diffusion-weighted images.

After a series of image processing, 76 features of generalized fractional anisotropy and 76 features of mean diffusivity values were obtained from two-shell diffusion-weighted images, and these processed image features of the training set were used to create a brain age prediction model. The sex factor was also included as a predictor in the models. The neuroimaging features of the WM-based model input consisted of 152 features, respectively. Twelve-layer feed-forward cascade neural network models, which provide an accurate prediction with flexible model architecture, were used to predict age (Chen et al., 2020). The cascade neural network is a feed-forward neural network involving connections from the input and every previous layer to the subsequent layer. This network is similar to a simplified fully connected version of a dense block in densely connected convolutional networks, which avoid the vanishing-gradient problem and strengthen feature propagation (Huang et al., 2017). The loss function of model optimization was specified as a mean square error function, which was optimized using a gradient descent algorithm with an adaptive learning rate and constant momentum. A 10-fold cross-validation procedure was performed to estimate the performance of the brain age model in the training phase. Next, an independent test set was used to evaluate the generalizability of the models. The modeling procedures were implemented using MATLAB R2019a (MathWorks Inc., Natick, MA, USA) with an NVIDIA GeForce RTX 2080Ti (NVIDIA Inc., Santa Clara, CA, USA) graphics processing unit for accelerated computing. The model performance was evaluated according to the Pearson correlation coefficient and mean absolute error between the estimated brain age and chronological age. Finally, the WM-based brain age prediction model were ready to predict brain age measures in the target set. The demonstration code of brain age modeling is available for open-access in the author’s github repository (https://github.com/ChangleChen/BrainAge_TL).

S1.2 Modeling approach selection for brain age regression

Algorithm choice of brain age regression varied in the previous studies. Several state-of-the-art algorithms including support vector machine (SVM), Gaussian process regression (GPR), Ensemble learning, and artificial neural network are preferable choices and have been utilized to create brain age models (Cole et al., 2019). To determine which algorithm was most satisfactory for brain age modeling, before the current study, we conducted an internal test to evaluate different modeling approaches. Given the feature sources and the imaging process method used in the present study, we proposed several suitable and popular algorithms, namely SVM, GPR, Ensemble learning, and deep neural network, to train brain age models. All the machine learning methods are implemented using MATLAB R2018b.

The model specifications are shown below:

1. Model 1: SVM method with linear kernel function and sequential minimal optimization.
2. Model 2: SVM method with radial basis function (RBF) kernel and sequential minimal optimization.
3. Model 3: Ensemble model based on the regression tree, with the least-square boosting ensemble-aggregation method. The maximal number of decision splits per tree is 15 and the number of ensemble learning cycles is 500.
4. Model 4: Ensemble model based on the regression tree, with the bootstrap aggregating (bagging) method. The maximal number of decision splits per tree is 15 and the number of ensemble learning cycles is 500.
5. Model 5: GPR method with exponential kernel and quasi-newton optimizer.
6. Model 6: GPR method with rational quadratic (RQ) kernel and quasi-newton optimizer.
7. Model 7: Cascade neural network (CasNN) model, introduced in the main text and S1.1.

The training and test sets constituted 500 and 116 observations, respectively, from the CamCAN cohort with the advanced features. Details of the data description were mentioned in the main text. The model performance evaluation was measured quantitatively using 1) Pearson correlation coefficient (PCC) between predicted age and chronological age, and 2) mean absolute error (MAE) between predicted age and chronological age. Ten-fold cross validation was used to assess model accuracy in the training set. The confidence intervals of the evaluated metrics were estimated by 1,000 bootstrap samples.

In our internal test, the CasNN method outperformed other competitors based on the model performance in the training and test sets. Therefore, we adopted the CasNN method to train the brain age prediction model.

The results of brain age regression by different approaches:

ML Methods for regression	PCC @Training	MAE @Training	PCC @Test	MAE @Test
Model1 (SVM, Linear)	0.891 (0.868,0.914)	6.85 (5.92,7.79)	0.869 (0.853,0.885)	7.66 (7.20,8.12)
Model2 (SVM, RBF)	0.912 (0.888,0.936)	6.04 (5.18,6.91)	0.879 (0.863,0.895)	7.28 (6.84,7.72)
Model3 (Ensem., LSBoost)	0.813 (0.766,0.860)	8.89 (7.65,10.13)	0.752 (0.722,0.782)	9.77 (9.17,10.37)
Model4 (Ensem., Bagging)	0.878 (0.844,0.912)	7.08 (6.03,8.13)	0.851 (0.826,0.876)	7.79 (7.23,8.35)
Model5 (GPR, Expon.)	0.932 (0.916,0.948)	5.38 (4.52,6.24)	0.916 (0.903,0.929)	5.88 (5.46,6.30)
Model6 (GPR, RQ)	0.930 (0.915,0.945)	5.44 (4.61,6.28)	0.918 (0.907,0.929)	5.95 (5.54,6.36)
**Model7 (CasNN)**	**0.958 (0.952,0.964)**	**4.16 (3.97,4.36)**	**0.943 (0.941,0.945)**	**4.68 (4.59,4.77)**

References:

Chen C.L., et al. (2020). Generalization of diffusion magnetic resonance imaging-based brain age prediction model through transfer learning. Neuroimage 217:116831.

Cole, J. H., et al. (2019). Quantification of the Biological Age of the Brain Using Neuroimaging. In Healthy Ageing and Longevity (pp. 293–328). Springer International Publishing. https://doi.org/10.1007/978-3-030-24970-0_19

Huang, G., et al. (2017). Densely connected convolutional networks, in Proceedings of the IEEE Conference on Computer Vision and Pattern Recognition. pp. 2261–2269.
